# Supplementary material for: Optogenetic control of excitatory post-synaptic differentiation through neuroligin-1 tyrosine phosphorylation
Source: eLife. 2020 Apr 23;9:e52027. doi: 10.7554/eLife.52027 (PMC7180054; doi:10.7554/eLife.52027)
Supplement: Source code 1. [file elife-52027-code1.docx]

**Program description**

This Mathematica program simulates individual AMPAR trajectories within a 2D geometry representing a typical dendritic segment of width 2 µm and length 10 µm, containing 5 synapses (dimensions 0.3 x 0.3 µm, spaced every µm). The trajectory length is n= 21000 steps of 100 ms (35 min). AMPARs alternate between periods of free diffusion in extra-synaptic compartments (‘outside’), and periods of slower diffusion when they penetrate in synapses (‘inside’). When inside a synapse, they can interact transiently with quasi-immobile post-synaptic scaffolding elements of size 0.15 x 0.05 µm(‘bound’). The transitions between free and bound states are governed by 2 kinetic rates (kc and ku, inverse of seconds). After a baseline of 5 min, the dissociation rate is lowered in the central synapse to mimick LTP. The output of the program is a text file (sortie.dat) with 6 columns (simulation number, frame number, x-coordinate (µm), y-coordinate (µm), area (10 pixels), intensity (1000 arbitrary units)). It is saved by default in the Mathematica folder. Before excecuting the program, please remove the notes in blue.

**********************************************************************************

<< Statistics`ContinuousDistributions`

ndist = NormalDistribution[0, 1];

*Dimensions of the simulations

n = 21000;

delta = 0.1;

timezero = 3000;

nsimul = 1;

step = 0;

*Diffusion coefficients

outside = 0.10;

inside = 0.05;

psd = 0.006;

free = Sqrt[2*outside*delta];

cluster = Sqrt[2*inside*delta];

bound = Sqrt[2*psd*delta];

*Kinetic rates

kc = 1.0; ku = 0.04;

coupl = kc*delta;

detach = ku*delta;

koff = 0.004;

*Dendritic segment geometry

a = 0.15; d = 0.3;

b = 2;

espace = 5;

c = IntegerPart[espace/b];

width = 2;

*Data file definition

fichier = Table[{1, 1, 5, 1, 10, 1000}, {n*nsimul}];

*Simulation loop

nombre[0] = Random[];

For[i = 1 + step, i < nsimul + 1 + step, i++,

Print[i];

*Initial conditions

xexo = 2*espace*(Random[] - 0.5);

ygrec0 = width*(Random[] - 0.5);

ltp = ku*delta;

absc = Table[xexo, {n}];

ord = Table[ygrec0, {n}];

graphe = Table[{xexo, ygrec0}, {n}];

For[j = 1, j < n + 1, j++,

synapse = False;

binding = False;

potentiation = False;

x[1] = xexo;

y[1] = ygrec0;

numen = Random[];

x1 = Random[ndist];

y1 = Random[ndist];

If[j > timezero, ltp = koff*delta];

If[-a < x[j - 1] < a \[And] -a < y[j - 1] < a \[And] coupl > nombre[j - 1] ,

While[x[j - 1] + bound*x1 < -a \[Or] x[j - 1] + bound*x1 > a,

x1 = Random[ndist];];

x[j_] = x[j - 1] + bound*x1;

While[y[j - 1] + bound*y1 < -a \[Or] y[j - 1] + bound*y1 > a,

y1 = Random[ndist]];

y[j_] = y[j - 1] + bound*y1;

absc = ReplacePart[absc, x[j], j];

ord = ReplacePart[ord, y[j], j];

potentiation = True;

If[ltp < numen, nombre[j_] = nombre[j - 1], nombre[j_] = Random[]];

];

If[potentiation == False,

For[k = -c, k < c + 1, k++,

If[k != 0 \[And]

k*b - a < x[j - 1] < k*b + a \[And] -a < y[j - 1] < a \[And]

coupl > nombre[j - 1] ,

While[x[j - 1] + bound*x1 < k*b - a \[Or]

x[j - 1] + bound*x1 > k*b + a, x1 = Random[ndist];];

x[j_] = x[j - 1] + bound*x1;

While[y[j - 1] + bound*y1 < -a \[Or] y[j - 1] + bound*y1 > a,

y1 = Random[ndist]];

y[j_] = y[j - 1] + bound*y1;

absc = ReplacePart[absc, x[j], j];

ord = ReplacePart[ord, y[j], j];

binding = True;

If[detach < numen, nombre[j_] = nombre[j - 1],

nombre[j_] = Random[]];

];

If[binding == True, Break[]];

];

];

If[binding == False \[And] potentiation == False,

For[k = -c, k < c + 1, k++,

If[k*b - d < x[j - 1] < k*b + d \[And] -d < y[j - 1] < d,

x[j_] = x[j - 1] + cluster*x1;

y[j_] = y[j - 1] + cluster*y1;

absc = ReplacePart[absc, x[j], j];

ord = ReplacePart[ord, y[j], j];

synapse = True;

nombre[j_] = Random[];

];

If[synapse == True, Break[]];

];

];

If[binding == False \[And] synapse == False \[And] potentiation == False,

If[Abs[x[j - 1] + free*x1] < espace, x[j_] = x[j - 1] + free*x1,

If[x[j - 1] + free*x1 > espace ,

x[j_] = 2*espace - x[j - 1] - free*x1];

If[x[j - 1] + free*x1 < -espace,

x[j_] = -2*espace - x[j - 1] - free*x1]];

absc = ReplacePart[absc, x[j], j];

If[Abs[y[j - 1] + free*y1] < width/2, y[j_] = y[j - 1] + free*y1,

If[y[j - 1] + free*y1 > width/2 ,

y[j_] = width - y[j - 1] - free*y1];

If[y[j - 1] + free*y1 < -width/2,

y[j_] = -width - y[j - 1] - free*y1]];

ord = ReplacePart[ord, y[j], j];

nombre[j_] = Random[];

];

graphe = ReplacePart[graphe, {Extract[absc, j], Extract[ord, j]}, j];

fichier =

ReplacePart[

fichier, {i, j, 5 + Extract[absc, j], 1 + Extract[ord, j], 10,

1000}, (i - 1 - step)*n + j];

];

*Plot of the trajectory

ListPlot[graphe, PlotJoined -> True, AspectRatio -> Automatic,

PlotRange -> {{-espace - 1, espace + 1}, {-width/2, width/2}}];

];

*Data export

Export["sortie.dat", fichier, "Table"];
